# Clustering of Neural Activity: A Design Principle for Population Codes

**DOI:** 10.3389/fncom.2020.00020

**Published:** 2020-03-13

**Authors:** Michael J. Berry, Gašper Tkačik

**Affiliations:** ^1^Princeton Neuroscience Institute, Princeton University, Princeton, NJ, United States; ^2^Institute of Science and Technology Austria, Klosterneuburg, Austria

**Keywords:** population coding, maximum entropy, information theory, correlations, clusters, error correction, unsupervised learning, criticality

## Abstract

We propose that correlations among neurons are generically strong enough to organize neural activity patterns into a discrete set of clusters, which can each be viewed as a population *codeword*. Our reasoning starts with the analysis of retinal ganglion cell data using maximum entropy models, showing that the population is robustly in a frustrated, marginally sub-critical, or *glassy*, state. This leads to an argument that neural populations in many other brain areas might share this structure. Next, we use latent variable models to show that this glassy state possesses well-defined clusters of neural activity. Clusters have three appealing properties: (i) clusters exhibit *error correction*, i.e., they are reproducibly elicited by the same stimulus despite variability at the level of constituent neurons; (ii) clusters encode qualitatively *different visual features* than their constituent neurons; and (iii) clusters can be learned by downstream neural circuits in an *unsupervised* fashion. We hypothesize that these properties give rise to a “learnable” neural code which the cortical hierarchy uses to extract increasingly complex features without supervision or reinforcement.

## Introduction

Throughout the central brain, information about the external world, internal body states, and movement plans is represented by large populations of neurons. The code employed by such neural populations has been the subject of extensive and ongoing study. Because nearby neurons typically exhibit significant correlation in their activity, their population code is necessarily combinatorial, in the sense that the message conveyed by the activity of one neuron is modified by the activity of nearby, correlated neighbors. Although the pairwise correlations between neurons typically are weak, these correlations can have a strong effect on the probability of population activity patterns (Schneidman et al., 2005, 2006). This implies that the principles that operate at the level of population codes may be significantly different from those that are evident for small groups of neurons.

A number of such population coding principles have been identified. First, correlated population codes can have a fidelity that is very different from matched, independent codes (Panzeri et al., 1999; Wilke and Eurich, 2002; Shamir and Sompolinsky, 2006; da Silveira and Berry, 2014; Kohn et al., 2016). In particular, positive noise correlations that have been observed experimentally can substantially reduce and limit discrimination performance (Zohary et al., 1994; Sompolinsky et al., 2001). In other cases, neurons can have patterns of correlation that can increase their mutual information about stimuli (Tkačik et al., 2010; Franke et al., 2016; Zylberberg et al., 2016). In general, mutual information is decreased when signal and noise correlations have the same sign (Oram et al., 1998; Schneidman et al., 2003a; Josic et al., 2009; Moreno-Bote et al., 2014), and this pattern seems most commonly observed experimentally (Cohen and Kohn, 2011).

Second, population codes can simultaneously represent not just an estimate of a sensory or motor variable, but also the entire probability distribution over the occurrence of that variable (Zemel et al., 1998; Pouget et al., 2000; Orban et al., 2016; Aitchison and Lengyel, 2017). Representation of entire probability distributions is favorable for carrying out Bayesian inference (Ma et al., 2006; Beck et al., 2008). Finally, we also note that individual neurons can multiplex different kinds of information into different spike train variables (Victor and Purpura, 1998; Meister and Berry, 1999; Lundstrom and Fairhall, 2006). For instance, individual spikes can convey a local estimate of the stimulus while the time interval between spikes can represent the contrast of the stimulus ensemble (Lundstrom and Fairhall, 2006). Evidence for temporal multiplexing also exists at the population level (Lankarany et al., 2019).

Another broad coding principle is the idea that neural codes should be sparse. This principle has been used to explain the organization of receptive fields in the primary visual cortex (Olshausen and Field, 1996) and other sensory pathways (Lewicki, 2002; Hyvärinen et al., 2009; Blattler and Hahnloser, 2011). This coding principle is consistent with experimental results from many brain areas, in which the activity of most projection neurons (such as pyramidal cells in the neocortex) tends to have a low probability of spiking in a small timebin as well as a skewed distribution of spike rates with a long tail (Baddeley et al., 1997; Buzsaki and Mizuseki, 2014). Sparse coding is also linked to statistically optimal linear encoding (“independent component analysis” or ICA) of natural stimuli with a sparse generating structure (Bell and Sejnowski, 1995). While sparseness as a coding principle is associated with a reduction in redundancy among neurons, it need not achieve full statistical independence to be a useful coding principle. Furthermore, sparseness connects to ideas about energy efficiency, as action potentials and synaptic currents account for a large fraction of the brain’s energy balance (Attwell and Laughlin, 2001; Lennie, 2003). As we shall see below, sparseness is consistent with our proposed design principle.

Sparseness is closely related to one of the most popular design principles: efficient coding. We can distinguish three kinds of efficient coding principles. Historically, the first such principle was redundancy reduction, proposed to explain the center-surround organization of retinal receptive fields (Attneave, 1954) – an idea that was generalized to central brain circuits (Barlow, 1961; Dan et al., 1996). A second, more general version of redundancy reduction is the principle that the population code should be as close as possible to the channel capacity (Atick, 1992; Atick and Redlich, 1992), an organizing principle closely related to “infomax” (Linsker, 1988). A third principle is predictive coding, an implementation of redundancy reduction which assumes that neural codes use regularities in the environment to emphasize surprising sensory information and hence improve coding efficiency. This idea was first proposed for the retina (Srinivasan et al., 1982) and later generalized to the cortical hierarchy (Mumford, 1992; Rao and Ballard, 1999; Bastos et al., 2012). Fourth, and related to predictive coding, is the idea that local circuits carry out computations that selectively or optimally encode predictive information (Bialek et al., 2001; Palmer et al., 2015), which can be interpreted as a more general optimization principle that entails redundancy reduction in a low noise limit (Chalk et al., 2018).

A major challenge to efficient coding is the fact that real population codes have high redundancy (Barlow, 2001; Diamond et al., 2003; Narayanan et al., 2005; Puchalla et al., 2005). Related is the fact that the activity of one neuron can often be accurately predicted from the activity of its neighbors (Tkačik et al., 2014). Therefore, the classic version of redundancy reduction can be ruled out empirically. While redundancy can optimize the encoded information in a high noise limit (Tkačik et al., 2010), retinal redundancy appears to be considerably higher. For instance, one study found that a mosaic of ganglion cells with ∼10% redundancy optimally encoded information (Borghuis et al., 2008), while the entire population has a redundancy of ∼11-fold (Puchalla et al., 2005). Regardless, most neuroscientists share the intuition that population codes are likely to be “well designed.” These considerations suggest that there are other benefits to redundancy, beyond mere noise suppression; in this paper, for example, we put forward the hypothesis that “learnability” is one such benefit. Perhaps population codes may someday be appreciated to be optimal or nearly optimal, once a larger and more realistic set of constraints on their structure have been considered.

### Overview

In this paper, we review evidence for a new design principle – namely, that population neural activity is robustly organized into clusters. This evidence primarily comes from retinal ganglion cells, but also lends itself to an argument that this property extends to many, if not most, neural populations in the central brain. We lay out the basic logic of our argument as follows (with the details contained in subsequent sections). First, we analyze the retinal ganglion cell population using maximum entropy models to closely approximate their probability landscape. By exploiting analogies between the maximum entropy model and the Boltzmann distribution in statistical physics, we show that the population is in a marginally sub-critical state, with frustrated interactions between constituent neurons. We call this a *glassy* state. We find that the glassy state is robustly present for a wide variety of stimulus ensembles, both artificial and natural, as well as across adaptation states. Because the properties of the maximum entropy model depend only on low-order statistics of neural activity, like firing rates and pairwise correlations, any neural population with similar low-order statistics will be in the same glassy state. We show that the retinal ganglion cell population has pairwise correlations that are strong enough to robustly realize such a state. This leads to the implication that many neural populations in the brain are likely to share this common structure.

**FIGURE 1:**
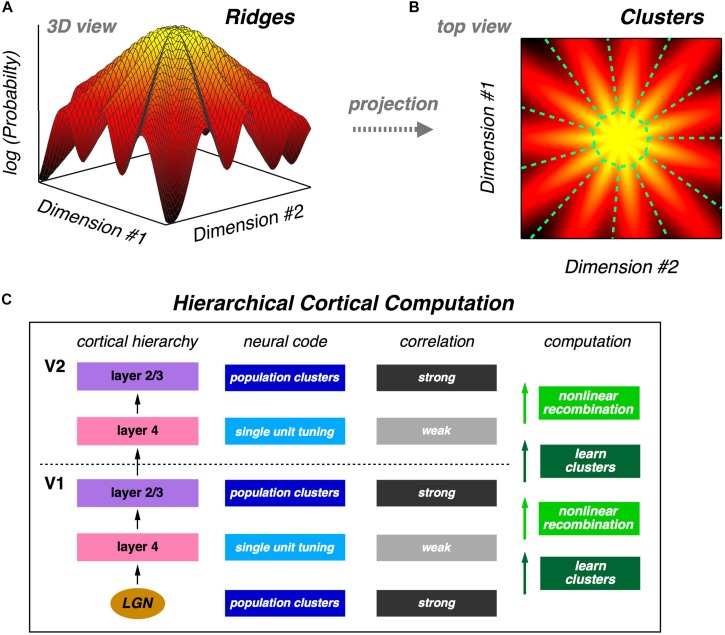
Population neural activity is organized into discrete clusters. **(A)** Schematic illustration of the global organization of population neural activity; the probability landscape resembles a mountain with a set of ridges descending from the summit. **(B)** Top view of the same probability landscape; each ridge can be viewed as a cluster of neural activity (demarcated by dashed green lines). **(C)** Proposal for hierarchical feature learning. Clusters are learned in layer 4 of the neocortical microcircuit, allowing layer 4 neurons to represent new sensory features with reduced correlation. Layer 2/3 generates nonlinear recombinations of layer 4 neural activity, thus creating a new population code with stronger correlation and new clusters of neural activity. These signals propagate up the cortical hierarchy, and the next layer 4 can again learn clusters in its input population, which are different than the lower-level clusters. This alternating process of cluster learning followed by recombination to form new clusters can repeat going up the cortical hierarchy, generating selectivity to increasingly complex sensory features.

The fact that a neural population is in a glassy state is a statement about the qualitative structure of its probability landscape. Specifically, this result suggests that there might be many local peaks in that landscape. Following this intuition, we analyze the population using latent variable models, which also match neural activity statistics very well. In these models, each latent state corresponds to a *cluster* of neural activity, which then defines a mapping from any neural activity pattern onto a unique cluster. We show that clusters are well-separated and reliably activated by the same visual stimulus, thus exhibiting a form of error correction. We also show that the receptive fields of clusters are, in general, qualitatively different from the receptive fields of individual neurons. Then, we explore the geometry of the probability landscape of neural activity patterns by moving locally on that landscape; this analysis reveals that clusters typically have the geometry of a *ridge*, not a local peak. On a global scale, we find that the entire probability landscape resembles a mountain with the all-silent state at the summit and ridges radiating downwards in different directions (Figures 1A,B).

The set of neural activity patterns that map onto the same cluster typically have a simple structure. There is an “active set” that is a small subset of all of the neurons. Within the active set, a threshold number of neurons must have a spike. All of the other neurons are in the “silent set” and thus must be silent. This structure closely resembles Hebb’s idea of cell assemblies (Hebb, 1949). In addition, this simple structure allows for clusters to be learned in an unsupervised fashion. We show that a winner-take-all (WTA) circuit with Hebbian plasticity in its feedforward synapses can learn clusters in the retinal ganglion cell population. The computational ingredients of this WTA circuit are quite generic in the brain, thus implying that many local circuits have the ability to make use of the clustered structure of neural population codes.

The organization of the population code into clusters suggests a powerful design principle which we call “learnability.” A good population code might not be information-efficient, but instead it could have statistical properties that allow downstream neural circuits to learn such a code in a manner that is unsupervised and sample efficient. If we take the perspective of a downstream neural circuit, its input is, of course, a stream of population neural activity. If those activity patterns are organized into clusters, then the obvious (perhaps even inevitable) computation for that neural circuit to carry out is to learn those clusters. And because clusters represent qualitatively different visual features, this unsupervised learning *automatically* teaches the downstream circuit about useful distinctions in the stimulus. What is more, ascending pathways in the neocortex lend themselves to a hierarchical version of cluster formation (Figure 1C) – an idea that we return to below.

### Neural Coding: Encoding Models Versus Activity Models

Neural activity is noisy, in that the same synaptic inputs will result in different trial-to-trial spiking outputs. As a result, the neural code is necessarily probabilistic. Sets of sensory or motor events, denoted by *S*, are thus related to population neural activity, denoted by *R*, through the joint probability distribution, *P*(*R*,*S*). Full knowledge of this distribution would constitute a solution to the neural code. However, in practice, this is rarely achievable. One approach is to focus on the conditional probability, *P*(*R*|*s*), where *s* is a single stimulus. For individual neurons, this is typically known as a tuning curve for a small set of {*s*} or receptive field model for larger sets of {*s*}. This approach, which we denote the *encoding model*, is common and quite intuitive: one presents a set of stimuli to neurons, records their responses, and then uses a variety of quantitative methods to construct general models linking the two. To the extent that such models accurately capture the responses of many neurons over a broad set of stimuli, then this version also constitutes a solution to the neural code. While there are notable examples of cell types and stimulus sets for which such models are highly successful, it is unclear how well this approach will work for the full set of behaviorally relevant stimuli and whether it correctly captures the correlation structure present in the neural population (Rodieck, 1965; van Hateren et al., 2002; Pillow et al., 2008; Chen et al., 2013; McIntosh et al., 2016; Buckley and Toyoizumi, 2018).

But regardless of the success of encoding models, this approach is not well matched to the way that the brain works. The reason is that in this approach, neuroscientists must select a set of stimuli that are entirely known. Neural circuits in the brain do not have access to “ground truth” stimuli and never perfectly know the states of the external world – that is the job the entire sensory system to estimate. Next, neuroscientists endeavor to measure as comprehensively as possible the response of a neural population. Experimental limitations often result in approximations, such as attempting to predict the firing rate of individual neurons. However, in the brain, downstream neural circuits always get the complete, simultaneous neural activity patterns – by definition – and never their trial-averages. Finally, the goal of model construction is to predict the neural response as accurately as possible. But again, the brain never needs to make such predictions, because downstream circuits automatically receive the true population activity. Instead, the brain uses many samples of population neural activity to make increasingly accurate estimations of behaviorally-relevant stimuli or their latent causes. Having some form of implicit knowledge of receptive field functions of input neurons might be useful in this task, but it is not required.

With these ideas in mind, we have taken a different approach, that we denote the *activity model.* In this approach, we focus instead on the properties of the probability distribution accumulated over an entire stimulus ensemble, $P\,\left(R\right)=\sum_{s}^{s\,t\,i\,m\,u\,l\,i}P\,\left(R|s\right)\,P\,\left(s\right)$. The goal is to understand the structure of this distribution. One obvious benefit of this approach is that it more closely matches the actual task that the brain must solve. In addition, there is no requirement to have successful receptive field models. Instead, one simply measures the true population activity. Furthermore, one need not define cell types or subsets in the neural population, as all that matters is the joint statistics of neural activity. One drawback of this approach is that the answer one gets depends fundamentally on the stimulus ensemble. However, the success of encoding models can also depend on the stimulus ensemble. For instance, the classic linear-nonlinear (LN) model developed by Rodieck works for objects moving smoothly on the retina (Rodieck, 1965), but when objects reverse direction an LN-LN cascade with gain control is needed (Chen et al., 2014). Similarly, when there is wide-field object motion, a new form of inhibition from wide-field amacrine cells comes into play (Olveczky et al., 2003). This dependence is less direct and clear for encoding models than for activity models, but is still present nonetheless.

### Retinal Origins of the Design Principle

This paper focuses on neural data and analysis from the retina. Most readers will find our perspective on the population code of retinal ganglion cells to be unfamiliar and perhaps counter-intuitive. The better-established framework for understanding visual coding in the retina is the parallel channels view (Werblin et al., 2001; Masland, 2012). This view emphasizes the role of different types of retinal ganglion cells. Each cell type has dendrites, and hence spatial receptive fields, that efficiently cover, or tile, the area of the retina, and hence visual space. Each ganglion cell type receives specific inputs from different types of retinal interneurons that together constitute distinct microcircuits that give rise to specific feature selectivity in that ganglion cell type. Ganglion cell types are often given evocative names – such as ON-OFF direction selective (Barlow et al., 1964), object-motion selective (Olveczky et al., 2003), or local edge detector (van Wyk et al., 2006) – that give a qualitative description of the visual features that best trigger them. In this view, the primary project for future retinal research is to define and delineate the different cell types (interneurons as well as ganglion cells), determine the synaptic contacts in their different microcircuits, and fill out the entire set of visual feature selectivity.

While this research program is clearly valuable, it fails to address a crucial topic: How do the ganglion cells encode information as an *entire population*? In the parallel channels view, each ganglion cell type communicates to the brain an entire “image” of visual space, filtered by its receptive field function. However, there is no principle or unifying idea that describes how these different images interact. One possibility is that different ganglion cell types project to different brain centers, each carrying out a specific and understandable function. Clearly, this is true for some ganglion cell types, such as ON direction selective cells that project to the accessory optic system and provide retinal slip information for the vestibular-ocular reflex (VOR) or the M1 melanopsin-containing cells that project to the suprachiasmatic nucleus to indicate the overall light intensity of the world (Dhande et al., 2015). However, the dominant targets in the central brain – the superior colliculus and the thalamus – receive inputs from many ganglion cell types. For instance, in the rabbit, 97% of all ganglion cells project to the superior colliculus (Vaney et al., 1981). In the macaque monkey, at least 13 cell types project to the LGN (Dacey et al., 2003). Thus, there is an extensive population code in the dominant visual pathways.

Another related question is: Why are there so many retinal ganglion cell types? The actual number of cell types has not yet been finalized. In fact, the number reported in the literature has grown over time. The most current estimate is 32–36 in the mouse (Baden et al., 2016), and connectomics data suggest that the number might be 40 (Bae et al., 2018). Current theories or design principles typically do not predict this great diversity of ganglion cell types. The efficient coding hypothesis, as proposed by Barlow (1961) and developed by Atick (Atick, 1992; Atick and Redlich, 1992) only describes how the receptive fields of a single cell type should be organized. Recent extensions have managed to predict the emergence of more than one type, but are still far from accounting for the observed diversity (Gjorgjieva et al., 2014; Sharpee, 2017; Ocko et al., 2018).

We can address these questions using activity models, as we will see below. But first, one key issue that we must address in this approach is to determine the size of population coding unit and then measure the activity of such coding units. Fundamentally, this population size is set by the scale of correlations between neurons. If two cells possess correlation, then the message encoded by the spike of one cell depends on whether the second cell is spiking or silent. Thus, there exists a combinatorial population code among correlated neurons. In the retina, neighboring ganglion cells of the same type typically exhibit significant correlation (DeVries, 1999; Shlens et al., 2009). While the subject is not often reported, ganglion cells of different functional types with overlapping receptive fields also share significant correlation (Segev et al., 2006). The spatial scale of correlation between ganglion cells extends out to distances of ∼400 μm (Puchalla et al., 2005), which corresponds to ∼2 receptive field diameters (Segev et al., 2006). The number of ganglion cells in a circle of this size is 200–300 cells. Thus, the population coding unit of the retina is 200 or more ganglion cells. This number corresponds to another simple calculation. If we assume nearest neighbor correlation and a mosaic arrangement of spatial receptive fields, then each cell type contributes 7 cells to the population coding unit; multiplying by 30+ cells types results in 200+ neurons.

What this means is that any location in visual space is encoded by roughly this many ganglion cells. This encoding is convolutional, in the sense that a slightly displaced location will be encoded by many of the same cells and some new ones. The shift in perspective that activity models bring relative to encoding models is the focus on the emergent, qualitatively new properties of these population coding units that are not apparent in smaller groups of cells. The key questions are thus not about the mechanisms by which individual cells of various types get their stimulus tuning properties, but rather on what correlated and coordinated behavior these mixed population coding units exhibit. As we will see below, we propose that the large number of retinal ganglion cell types is needed to put the population code into the glassy state.

### Maximum Entropy Models and the Glassy State

Understanding the structure of probability distribution over ∼200 neurons is a daunting task. This is because identifying landscape features such as peaks and ridges in a high-dimensional discrete space is a hard problem, irrespective of whether the parameters of the probability landscape can be tractably learned from data. Even if we focus only on whether each neuron has a spike or silence in a small timebin, there are 2*^*N*^* possible neural activity patterns. This number is literally astronomical: for *N* = 200, we get ∼10^60^ possible activity patterns. Such large numbers have important implications. First, we cannot sample all of these patterns experimentally. Furthermore, this is not a limitation of our neuroscience experiments – it also applies to behaving animals. So whatever approximations neuroscientists make might be reasonable for the brain, too. Second, most individual patterns occur rarely. Even in a human who lives to the age of 100 years, most of these patterns will never occur, and a substantial portion will have occurred only once in a lifetime. Clearly, the brain cannot associate individual meanings with single patterns of ganglion cell activity; some kind of coarse-graining is required.

Our approach has been to seek a good approximation to the full probability distribution, *P*(*R*), that is tractable. Specifically, we have used the maximum entropy principle to measure statistics of neural activity that can be well-sampled, such as the average firing rate and pairwise correlations of all neurons, and find the probability distribution with maximum entropy subject to these constraints. This probability distribution includes as few “assumptions” as possible beyond the explicit constraints, and thus is as smooth as possible given the constraints. There exist excellent reviews of the technique and its motivation (Pressé et al., 2013; Nguyen et al., 2017), so we will not retread that ground. We will denote the pairwise maximum entropy (MaxEnt) model as $P_{M\,a\,x\,E\,n\,t}^{\left(2\right)}\,\left(R\right)$. If we then discretize spike trains in 20 ms timebins, truncating more than 1 spike per bin, *R* = {*r*_*i*_} with *r*_*i*_ = [0, 1], we get:

(1) $$P_{M\,a\,x\,E\,n\,t}^{\left(2\right)}\,\left(R\right)=\frac{1}{Z}\,\exp\left\{\sum_{i}^{c\,e\,l\,l\,s}h_{i}\,r_{i}+\sum_{j>i}^{p\,a\,i\,r\,s}J_{i\,j}\,r_{i}\,r_{j}\right\},$$

where the parameters, {*h*_*i*_, *J*_*ij*_} are Lagrange multipliers that must be numerically optimized for the model to match experimental data (Schneidman et al., 2006; Shlens et al., 2006; Tkačik et al., 2006; Maoz and Schneidman, 2017). This numerical optimization procedure is computationally intensive, but tractable for populations of over 100 neurons (Shlens et al., 2009; Ganmor et al., 2011; Tkačik et al., 2014).

Empirically, this pairwise model has proven to provide an excellent approximation to the sampled statistics of ganglion cell activity for population sizes up to *N* ∼ 40 cells of all types with overlapping receptive fields (Schneidman et al., 2006; Tkačik et al., 2006) and *N* ∼ 100 cells of the same functional type (Shlens et al., 2009). For larger populations of all cell types, higher-order interactions start to become important. But the maximum entropy principle is flexible, so adding a sparse set of additional constraints results again in very good fits to data (Ganmor et al., 2011; Tkačik et al., 2014). Neural populations in the cortex can also be closely approximated using the maximum entropy principle, although higher-order interactions may be more significant (Ohiorhenuan et al., 2010; Koster et al., 2014; Maoz et al., 2018). The overall intuition is that the pairwise approximation is improved by sparse neural activity, but that analyzing larger populations amplifies the importance of higher-order interactions (Schneidman et al., 2003b), especially those that directly affect the distribution of synchronous spiking activity (Tkačik et al., 2014; Mora et al., 2015; Okun et al., 2015; Shimazaki et al., 2015; Humplik and Tkačik, 2017).

In addition to providing a good approximation to the entire probability landscape of a neural population code, the maximum entropy model also gives rise to a hypothesis about the overall structure of this landscape. This hypothesis arises from the fact that the mathematical form of the probability distribution closely resembles the Boltzmann distribution in statistical physics (Tkačik et al., 2015). In particular, if we identify the argument of the exponential in Eq. 1 as defining an energy-like quantity, then the maximum entropy model is isomorphic^1^ to the Boltzmann distribution for *T* = 1:

(2) $$\begin{array}{c} P_{M\,a\,x\,E\,n\,t}^{\left(2\right)}\,\left(R;T\right)=\frac{1}{Z\,\left(T\right)}\,\exp\left\{-\frac{E\,\left(R\right)}{T}\right\}\\ \;\text{with}\;E\,\left(R\right)\equiv -\sum_{i}^{c\,e\,l\,l\,s}h_{i}\,r_{i}-\sum_{j>i}^{p\,a\,i\,r\,s}J_{i\,j}\,r_{i}\,r_{j} \end{array}$$

The form of the energy function in the pairwise MaxEnt model is the same as found in variants of the Ising model. In particular, the interaction parameters, {*J*_*ij*_}, that give the best fit to neural data have both positive and negative values, giving rise to “frustration” (Schneidman et al., 2006). Since their distribution for *N* > 100 resembles a Gaussian with zero mean (Tkačik et al., 2014), one could expect that MaxEnt models for neurons behave similarly to the Sherrington-Kirkpatrick model for spin glasses (Mezard et al., 1987), although several fundamental differences exist (Tkačik et al., 2006; Catellana and Bialek, 2014). Ising-like models have been studied in physics for decades, which opens up the possibility that we can gain new neuroscience insight from an analogy to the statistical physics of spin glasses. More specifically, these models all describe various kinds of phase transition. So, an important question is: What kind of “phase” is the real neural population in?

We can explore the statistical physics of this wider class of MaxEnt models with an effective temperature variable, *T*. Of course, none of these models correspond to real neural populations, except at *T* = 1. But by varying the effective temperature, we can see if the population exhibits signatures of a phase transition at some value of *T* = *T*^∗^. The goal of this analysis will be to gain insight into what phase the real neural population is in.

In statistical physics, phase transitions occur formally only for systems of infinite size (in the “thermodynamic limit”) where there is a divergence in a susceptibility, like the specific heat (Humplik and Tkačik, 2017). For real, finite systems, a signature of an incipient transition is a peak in the same susceptibility quantity that grows larger as the size of the system is increased. Thus, we calculate the specific heat of the neural population as a function of the effective temperature,

(3) $$C\,\left(T\right)\equiv \frac{\partial \left\langle E\right\rangle }{\partial T}=\frac{\left\langle {\left(\delta \,E\right)}^{2}\right\rangle }{T^{2}}=\left\langle {\left(\delta_{\log}\,\left(P\right)\right)}^{2}\right\rangle \;\text{where}\;\left\langle E\right\rangle \equiv \sum_{R}^{s\,t\,a\,t\,e\,s}E\,\left(R\right)\,P\,\left(R\right)$$

From inspection of Eq. 3, we can see that the heat capacity measures how wide a distribution of energies (or log-probabilities) are present in *P*(*R*). Thus, the heat capacity is large for a neural population with a long-tailed probability landscape, and is small for a population with an approximately normal distribution. Once the parameters, {*h*_*i*_, *J*_*ij*_}, have been optimized to describe neural data, then the formulas in Eq. 3 can be evaluated. [Although for *N* > 20 cells, Monte Carlo sampling of *P*(*R*) is still necessary due the large number of neural activity patterns (Tkačik et al., 2006, 2014)].

**FIGURE 2:**
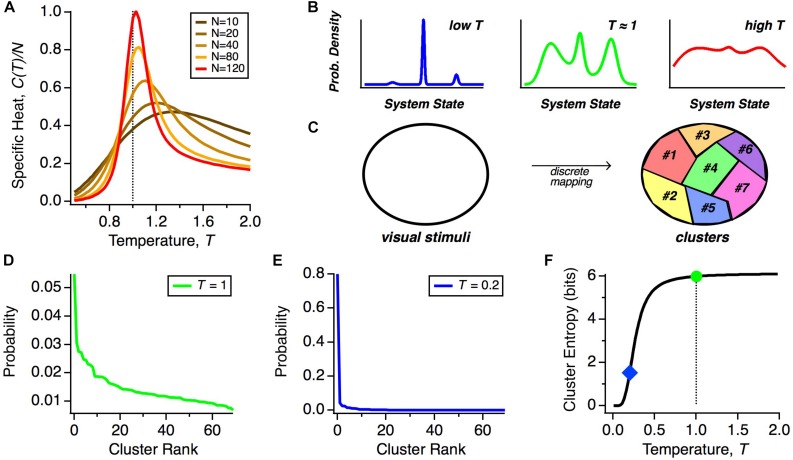
Thermodynamics of neural populations. **(A)** Specific heat (heat capacity per neuron, *C/N*) versus temperature (*T*) for neural populations of different sizes, *N*. **(B)** Schematic representation of the qualitative structure of probability distributions in different temperature regimes. **(C)** Schematic diagram of how stimulus space is divided into discrete classes by the mapping of population activity onto collective modes. **(D)** Probability distribution over clusters (shown in rank order) taken from real data (Prentice et al., 2016). **(E)** Schematic probability distribution over clusters at a lower temperature. **(F)** Schematic of the entropy of the cluster probability distribution as a function of temperature, *T*.

We can gain insight by exploring how the heat capacity depends on the number of neurons that we analyze. The reason is that because pairwise correlations are typically weak, their impact in shaping the population code is minimal for small *N* but can become more significant at larger *N* (Schneidman et al., 2006). Because the heat capacity increases with the number of neurons, *N*, we plot the specific heat, *C*(*T*)/*N*, to isolate non-trivial trends related to how correlations structure the population code. We see that as we increase the number of neurons analyzed together, the peak in the specific heat grows as a function of *N* neurons and shifts to lower effective temperatures (Figure 2A). This implies that the real state of large neural populations (*T* = 1 and *N* ∼ 200) shares properties with the state of a physical system poised near a phase transition.

Initially, we were struck by the fact that the peak in the heat capacity moved closer to *T* = 1 as we analyzed larger neural populations. This led us to speculate that the real system might be poised right at the peak, perhaps in a critical state (Mora and Bialek, 2011; Tkačik et al., 2015). However, there are several problems with this interpretation. First, a critical state exhibits long-range correlation, while measurements show that correlation between ganglion cells dies out at large enough spatial separation (DeVries, 1999; Puchalla et al., 2005; Segev et al., 2006; Pitkow and Meister, 2012). Second, the critical state is only truly defined in the thermodynamic limit of *N* → ∞, while the population coding unit for retinal ganglion cells is *N* ∼ 200.

These factors led us to develop a different interpretation: because the peak in the heat capacity was always above *T* = 1, any finite-sized neural population is instead in a *marginally sub-critical state*. Furthermore, the interaction parameters {*J*_*ij*_} that describe real neural data have a distribution with roughly equal numbers of positive and negative values (Tkačik et al., 2014), which results in *frustration* (Mezard et al., 1987; Schneidman et al., 2006). We call this frustrated, marginally sub-critical state the ***glassy*** state.

We want to emphasize that this terminology is not referring to the formal existence of a spin glass phase that could arise in specific statistical physics models in a thermodynamic limit – not the SK model (Mezard et al., 1987), nor the Hopfield network (Amit et al., 1985). The issue of whether such a phase could in principle be found in *inverse statistical physics* models is complicated (Catellana and Bialek, 2014). We also want to make clear that our model describes the *static properties* of the neural probability landscape and does not refer to any notion of slow dynamics. Specifically, the retina is a largely feedforward system, in which the state of ganglion cell population activity is driven by external stimuli. As such, the sequence of neural activity patterns that unfold across time are determined by the external stimulus. This sequence of activity thus, a priori, has nothing to do with the dynamics of a system moving on the energy landscape that we describe.

But what might this mean for the neural code? Because of the similarity of the solution for the optimal parameters of the MaxEnt model and the interaction parameters of spin glass models in physics, in particular, the frustration in the energy landscape (Tkačik et al., 2014), the distribution *P(R)* is expected to have many local maxima (energy minima). Far above the critical temperature, these local maxima of probability would be washed out, but once the system transitions below the critical temperature, as we observe, these peaks will become well-separated (Figure 2B). Our hypothesis is that all of the activity states within a single peak constitute a “cluster” of neural activity, which represents a single class of visual stimuli (Figure 2C). If we repeatedly present the same stimulus, the detailed activity pattern will vary, but we might find that those patterns reproducibly map onto the same cluster. Thus, these clusters would constitute population codewords that embody a representation of visual stimuli that is robust to neural noise.

However, we find that the system is not deep in the low temperature limit, but is instead poised near to, but below, the critical point. What is special about this operating point? If the neural population were too deep in the low temperature state, then the probability would be concentrated in just a few peaks, making the capacity of the cluster code low (Figure 2B, left). In this limit, a neural code that represented information based on the identity of the active cluster would have lower information capacity. We illustrate this point by showing a schematic of how the probability distribution over clusters, α, changes as a function of our temperature variable, *T*. We start with real data (Figure 2D) (Prentice et al., 2016). At *T* ≠ 1, we transformed this distribution according to *P*_*T*_(α)∼*P*_*T* = 1_(α)^(1/*T*)^. As seen for a significantly lower temperature, most of the probability weight shifted to the most common cluster (Figure 2E). As a result, the entropy of the cluster probability distribution dropped sharply in limit of sufficiently low temperature (Figure 2F).

The deep low temperature regime would also be inconsistent with experimental observations, since such putative sharp peaks would have to be smeared due to neural noise. In addition, the state of the retinal population changes smoothly in time, due to temporal integration of visual stimuli on a timescale ∼100 ms (Segev et al., 2006) that is larger than a single timebin in our analysis of the population code. This implies that as the population transitions between clusters, there will likely be one or more timebins in which the state is a mixture of those two clusters. Such smooth transitions will serve to blur the sharpness of each cluster. Thus, a cluster code that has high capacity and is consistent with data can be achieved as long as the system is in a low temperature state that is poised near to the critical point. Notice that this logic does not require that the neural population be tuned *exactly* to the critical state. Thus, we propose that the system should be designed to be poised near to the critical state in order to robustly constitute a high-capacity cluster code (Figure 2B, middle).

In this vein, we asked how the MaxEnt model fit to the data behaves if we scale up or down the neuron-neuron interactions (the *J*_*ij*_ terms in Eq. 2) while making sure that the neurons always spike with rates actually observed in the data (Tkačik et al., 2015). This manipulation results in independent neurons when the *J*_*ij*_ terms go to zero. By scanning the strength of the neuron-neuron interactions, we varied the strength of correlations away from the experimental values and observed three important effects. First, the neural population exhibited a peak in heat capacity close to the strength of the interactions (and hence correlations) present in real data, strengthening the evidence that the system is poised near to the critical point. Second, in the regime of correlations much stronger than in the data, we saw the emergence of a small number of strong clusters of spiking activity by simply looking at the rasters generated from the model. Third, the entropy remained roughly constant for correlations up to the observed values, but then decreased for stronger correlations. Together, these results are consistent with a probability landscape at lower temperatures that is disadvantageous for cluster coding (Figures 2B, left, E).

### Robustness of the Glassy State

**FIGURE 3:**
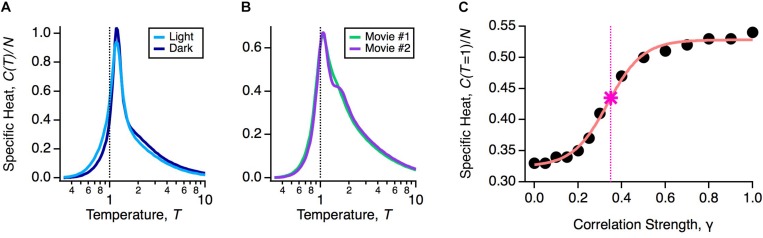
Robustness of the glassy state. **(A)** Specific heat versus temperature for the same neural population in the light (light blue) or dark (dark blue) stimulus conditions. **(B)** Specific heat versus temperature for the same neural population in two different natural movie ensembles. **(C)** Specific heat at *T* = 1 versus correlation strength (circles) with sigmoidal curve fit (pink) and critical correlation strength, γ^∗^ (star; vertical dashed line), defined as the midpoint of the sigmoidal fit.

If robustness is an important property of the population code, then it should be unaffected by natural variations in the functional state of the retina. The retina’s input-output function constantly changes during the day due to multiple mechanisms of adaptation. To explore the impact of these adaptive changes, we designed an experiment in which the same natural movie clip was shown at regular daylight conditions (light) or 1000-fold dimmer (dark). This is a rigorous test of robustness, because over this range of light intensities the retinal circuit switches between cone-dominated (photopic) and rod-dominated (scotopic) function (Ioffe and Berry, 2017). In fact, this change caused the average firing rates and pairwise correlations in the neural population to change significantly, indicating that the detailed population code differed across these two light levels (Ioffe and Berry, 2017). However, the heat capacity of the population was nearly identical (Figure 3A).

An essential consideration in using activity models is that the probability distribution will depend implicitly on the choice of stimulus ensemble. This is because even if there is no change in retinal function, different stimulus statistics will result in different firing rates and correlations among neurons. Furthermore, changes in statistics across different stimulus ensembles, such as contrast and spatial scale, can themselves trigger mechanisms of adaptation (Smirnakis et al., 1997; Hosoya et al., 2005). Thus, we wanted to test if the glassy state was robust across different choices of stimulus ensemble. To this end, we stimulated the retina with different natural movie clips (one of leaves blowing in the breeze, another of water in a river), again finding nearly identical heat capacities (Figure 3B). We also tested artificial stimulus ensembles, like flickering checkboards or spatially uniform flicker (Tkačik et al., 2014; Ioffe and Berry, 2017), and found that in all cases, the neural population was in a low temperature state poised close to criticality. These results have been reproduced in other labs, as well (Yu et al., 2013; Mora et al., 2015; Huang and Toyoizumi, 2016; Hahn et al., 2017).

To gain more insight into this robustness, we asked: by how much would we have to change the measured statistics of neural activity to transition out of the glassy state? Specifically, we formed model neural populations with all firing rates the same as our data and all pairwise correlations reduced by a common factor, *C*_*ij*_ → γ *C*_*ij*_. In the limit of γ → 0, we will have an independent neural population, which is in the high temperature state. For each value of γ, we optimized the parameters of the pairwise MaxEnt model and recomputed the heat capacity. We found that correlations needed to be scaled down by γ^∗^ ∼ 0.35 to transition to the high temperature regime (Figure 3C). In other words, the observed retinal correlations were roughly threefold stronger than needed to put the system in the glassy state.

This result gives rise to a powerful argument for the generality of the glassy state for neural populations outside of the retina. The properties of the MaxEnt model are entirely determined by the statistics of neural activity used as constraints, such as the firing rates and pairwise correlations. In other words, there is nothing in either the structure of the model or in the detailed activity statistics that applies only to the retina. Therefore, any neural population with equivalent statistics would also be in the glassy state. What counts as “equivalent statistics” is still under investigation (Catellana and Bialek, 2014), but clearly the average pairwise correlation strength and the population size are key aspects. This argument suggests that other neural populations with larger pairwise correlations than the retina or larger population coding units with similar correlation strength may also be in the glassy state.

### Latent Variable Models and Neural Activity Clusters

Our analyses of neural populations with the maximum entropy model imply that the retinal population exists in a state that is similar to a spin glass (Figure 2). This analogy suggests that the probability landscape is organized into a set of well-defined local peaks. If so, then another good model of *P*(*R*) would be a summation over functions that each represent one peak.^2^ Following on this intuition, we developed a hidden Markov model (HMM) as an alternative to the MaxEnt model, which is technically more favorable in terms of being able to fit the model’s parameters to data. The numerical tractability of this model allowed us to apply it to groups of over 200 ganglion cells as well as to incorporate temporal correlations. In this latent variable model, the probability distribution is modeled as a sum of individual terms labeled by α, where each term is an emission distribution or “mode”:

(4) $$\begin{array}{c} P_{H\,M\,M}\,\left(R\right)=\sum_{\alpha }^{m\,o\,d\,e\,s}w_{\alpha }\,Q_{\alpha }\,\left(R\right),\\ \;w\,h\,e\,r\,e\,Q_{\alpha }\,\left(R\right)=P_{\alpha }\,\left(r_{1}\right)\,\prod_{\left\langle i,j\right\rangle }^{e\,d\,g\,e\,s}P_{\alpha }\,\left(r_{i}|r_{j}\right) \end{array}$$

Each emission distribution, *Q*_α_, includes correlations between cells with a “tree” structure. This means that when we view the correlation structure as a graph, where each directed link is a conditional probability between the response of cells *i* and *j*, the links are not allowed to form loops (i.e., the graph is “acyclic”). This makes the model more tractable than the maximum entropy model, which does allow loops (Bethe, 1935; Baum, 1970). The intuition is that each distribution, α, captures one of the prominent peaks, or clusters, in the probability distribution *P*(*R*). In order to avoid overfitting, we determined the parameters of this model using cross-validation: iteratively solving for the parameters using two thirds of our data and testing performance on the remaining third. A key parameter of this model is the number of modes, *M*. We varied this parameter and selected the value with the highest cross-validated likelihood. Finally, we included temporal correlation with a simple Markov model form: *P*(α*_*t*_*_+__1_| α*_*t*_*). Such models were able to reproduce the experimentally sampled values of *P*(*R*) to within roughly the sampling noise for up to ∼200 ganglion cells (Prentice et al., 2016).

**FIGURE 4:**
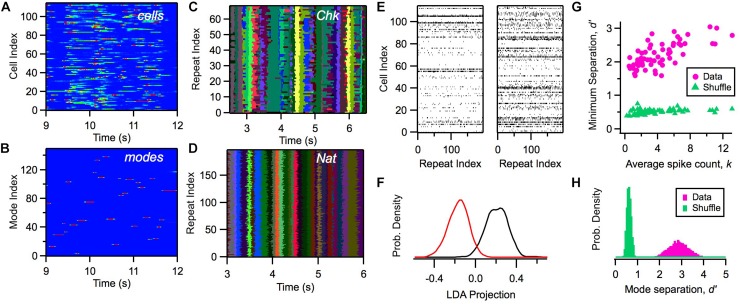
Defining clusters with a latent variable model. **(A)** Firing rate of 115 ganglion cells (color scale) versus time during a natural movie clip. **(B)** Probability of each mode occurring (color scale) versus time for the same movie as in panel **(A)**. **(C,D)** Most likely cluster across repeated presentations of (checkerboard flicker, natural movie) stimuli; different colors indicate different clusters. **(E)** Joint activity patterns across repeated presentations of a natural movie clip, presented for two different times in the movie (left, right); spikes shown in black. **(F)** Distribution of projections of activity states along a single direction in activity space defined by local discriminant analysis (LDA) for two clusters (red, black); these clusters are adjacent in response space. **(G)** Minimum separation (*d*′) between clusters for real data (pink) and a shuffle test (green) versus the average spike count in that cluster; each point is for a different cluster. In the shuffle test, we rearranged the firing rates within each cluster and re-optimized the rest of the model’s parameters. **(H)** Distribution of separation values (*d*′) for all cluster pairs for data (pink) and shuffle test (green).

Within this model, we can calculate the likelihood of a mode α*_*t*_* given a particular activity pattern in the ganglion cell population *R*_*t*_ and the previous mode α*_*t*_*_–__1_. Although the activity of individual cells exhibited a considerable range of firing rates distributed across time (Figure 4A), the likelihood of the activation of modes, *P*(α*_*t*_*| *R*_*t*_, α_*t* – 1_), was nearly binary in its values and exhibited sharp transitions between zero and one (Figure 4B). We mapped a given neural activity pattern, *R*^∗^, onto a unique cluster by finding the mode with the highest weighted probability, α^∗^ (Notice that clusters and modes use the same index; however, a cluster is a subset of all possible activity patterns, while a mode is a probability distribution defined over all *R*).

(5) $$\alpha^{*}\equiv \max_{\alpha }\left[w_{\alpha }\,Q_{\alpha }\,\left(R^{*}\right)\right]$$

This operation can also be seen as a Bayesian maximum-a-posteriori (MAP) decoding of the underlying mode from the noisy neural response, *R*^∗^; interestingly, here one infers the latent state of the population much as one would have inferred the stimulus from the response via the more traditional application of Bayes decoding to an encoding model (Quian Quiroga and Panzeri, 2009).

Due to the clear activation of individual modes across time, when we repeated the same natural movie clip many times, we found that the most likely cluster was essentially identical across stimulus repeats (Figure 4D). Interestingly, the activation of clusters was not as sharp and repeatable during stimulation with white noise (Figure 4C). This robustness of cluster activation is non-trivial, because noise caused the detailed activity pattern activated by the natural stimulus in a single time bin to vary greatly across trials (Figure 4E), yet all of these different activity patterns mapped onto the same cluster. This implies that the activation of clusters by a complex stimulus represents a robust coding variable for the neural population. In this sense, we say that clusters exhibit *error correction.*

We then proceeded to characterize the separation between different clusters: Are they really distinct peaks in the probability landscape, or are they just one possible partition of the responses without any clearly identifiable boundaries? We used linear discriminant analysis (LDA) to define a single direction in activity space that best separated the activity patterns mapped onto two different clusters. Then, we projected each activity pattern along the LDA dimension to yield a single number for each occurrence of a cluster. Even adjacent clusters were well separated (Figure 4F). We quantified this separation using *d*′, which is the difference in the mean of the two LDA projection distributions divided by the sum of their standard deviations; *d*′ > 1 is typically interpreted as “good separation.” Repeating this analysis, we found that all clusters had good separation (Figures 4G,H). This analysis provides direct evidence that the probability landscape of retinal population activity breaks up into well-separated clusters, as predicted for a system resembling a spin glass.

**FIGURE 5:**
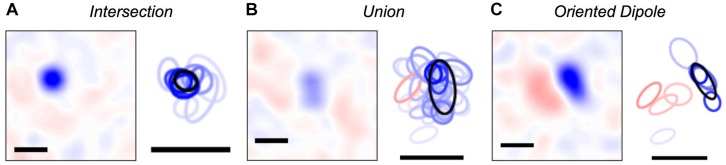
Visual features represented by clusters. **(A–C)** Left: Spatial profile of the cluster-triggered stimulus average during the checkerboard stimulus; (red, blue) is light intensity that is (above, below) the mean. Right: 1-standard deviation contour of a 2D Gaussian curve fit to the spatial profile for clusters (black) and individual cells (red = ON cells; blue = OFF cells; color saturation shows the firing rate of the cell within a given cluster). All scale bars are 340 μm. Example clusters show: **(A)** a smaller receptive field than its constituent neurons, **(B)** a larger receptive field than its constituent neurons, **(C)** ON and OFF subfields defining a preferred orientation.

Once we were able to map every activity pattern in the neural population onto a cluster, we could explore what were the set of visual stimuli represented by each cluster. To this end, we computed the cluster-triggered stimulus average during stimulation with random flicker. We found several qualitatively interesting cases. Some clusters were “intersections” that had a smaller spatial receptive field than any of the individual cells, allowing for greater spatial acuity than individual ganglion cells (Figure 5A); this result is an extension of previous findings for three cells (Schnitzer and Meister, 2003). However, other clusters were a “union” of individual ganglion cell receptive fields (Figure 5B). These clusters could be thought of as a position-invariant generalization of the trigger features of individual cells; such a process of spatial generalization occurs at many stages of the visual pathway (Riesenhuber and Poggio, 1999). Finally, some clusters had an “oriented dipole” spatial profile (Figure 5C). This result is especially intriguing, as orientation selectivity is not present among individual ganglion cells, yet it emerges in neurons in the primary visual cortex (V1). Perhaps neurons in the next stage of the visual pathway develop their tuning properties by “reading out” the identity of clusters?

### The Geometry of Clusters

**FIGURE 6:**
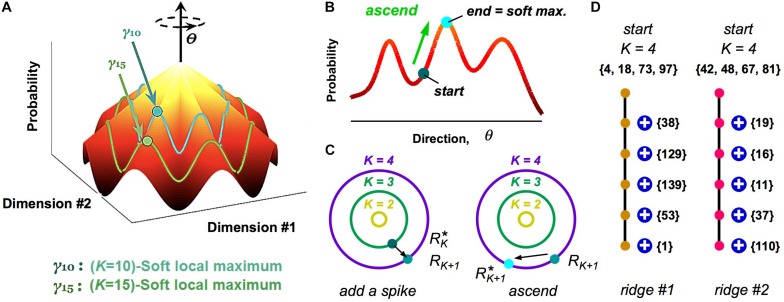
Visualizing the geometry of the probability landscape. **(A)** Schematic of the entire probability landscape. Radial coordinate is the number of spikes, *K*; angular coordinate, θ, represents a direction in activity space at constant spike count; *z*-axis is probability. Two subspaces of activity patterns at constant spike count are shown as colored lines (blue, green). Soft local maxima are denoted by circles. **(B)** Schematic illustration of the ascent procedure to find a soft local maximum. **(C)** Schematic illustration of how soft local maxima at successive spike counts are connected. Left*:* Starting with a soft max at *K*, you add a spike. Right: You then ascend to the nearest soft max at *K* + 1. **(D)** Enumeration of two example ridgelines. Starting with a *K* = 4 soft max (numbers denote neuron identity), connected soft local maxima at successively higher *K* mostly involve adding one spiking cell to the existing active set. Color of circles represents cluster identity.

While our analysis of clusters via a HMM is a powerful method to define population codewords and explore their properties, it still results in a somewhat abstract picture. To this end, we investigated the geometry of the probability landscape (Loback et al., 2017). This study led to a simple picture with which to visualize the entire probability landscape (Figure 6A): this landscape resembles a “mountain,” where the summit is the all-silent pattern (because neural activity is sparse). Descending from the summit in multiple directions in the space of neural activity patterns are different ridgelines. These *ridges* are the clusters.

To reach these conclusions, we first realized that clusters did not have the shape of a local peak in the probability landscape, at least not for the case of broad stimulus ensembles (Loback et al., 2017). This was discovered when we used a stochastic search procedure, where we randomly selected one neuron, changed its activity if that change increased the probability, and then iterated. This ascent procedure almost always mapped neural activity patterns to the all-silent pattern, as any point on the landscape ascends along a ridgeline to the summit (Figure 6A). Instead, we hypothesized that local peaks existed within the subspace of activity having the same spike count, *K*. To this end, we performed a search where we changed the activity pattern while keeping *K* fixed (Figure 6B); this procedure found many robust local peaks, which we called *soft local maxima* (Loback et al., 2017) (Here, “robust” means that we got the same answer over different stochastic search paths).

Next, we explored the organization of soft local maxima across different spike counts, *K*. To this end, we started in a soft max at *K*, changed one silent neuron to spiking, and then searched in the space of activity patterns with *K* + 1 spikes for the nearest soft local maxima (Figure 6C). In most cases, we identified a robust chain of connected soft local maxima, in which one spiking neuron was added at each increment of *K* (Figure 6D). This chain formed a ridgeline in the probability landscape. In other cases, a ridgeline terminated at *K*^*max*^, or split into two ridges. Finally, when we mapped soft local maxima onto the clusters defined by our HMM, we found that they corresponded to the same cluster (Figure 6D, colors). These analyses revealed that clusters have the geometry of a ridge – a highly non-Gaussian shape that would be difficult to discover with many clustering algorithms.

The nested structure of the set of soft local maxima forming a single ridge gave rise to a simple definition of which neural activity patterns map onto the same cluster. There is an “active set,” consisting of a subset of all neurons in the population (typically ∼15 out of 150 neurons). For the neural activity pattern to be in a given cluster, there can be a range of these neurons spiking, *K*^*min*^ ≤ *K* ≤ *K*^*max*^ (typical values, *K*^*min*^ = 4, *K*^*max*^ = 15). On the other hand, all the other neurons are part of a “silent set,” and *every one* of these neurons must be silent. Thus, silence of specific neurons makes the primary contribution to defining a cluster, while at the same time, the great tolerance for the number of spiking neurons helps give rise to error correction. This result generalizes earlier work showing that silence can significantly affect the meaning of a population activity pattern (Schneidman et al., 2011).

We also realized that the active set constitutes a *neuronal community*, a notion from graph theory. In addition, neuronal communities have most of the properties of *cell assemblies*, as defined by Donald Hebb. Thus, we realized that many different concepts about codewords in neural populations all refer to the same structure:

$$\begin{array}{c} \mathrm{Cluster}\approx \mathrm{Latent State of an HMM}\approx \mathrm{Ridge}\\ \;\approx \mathrm{Neuronal Community}\approx \mathrm{Cell Assembly} \end{array}$$

The confluence of these varied concepts about population codewords suggests that these different ideas are identifying different manifestations of a clear and conserved structure in the population code.

We should also note that in the case where the stimulus ensemble consists of many repeats of several different stimuli, the probability landscape instead is comprised of a local peak in probability corresponding to each discrete stimulus (Loback et al., 2017). This occurs because there is an average neural response that has relatively high probability with noise-corrupted versions at lower probability nearby. This dependence of the stimulus ensemble raises the interesting issue of how environmental context, perhaps mediated by feedback signals in the cortical hierarchy, might alter the processing of population codes.

### Learning and Reading Out Neural Activity Clusters

**FIGURE 7:**
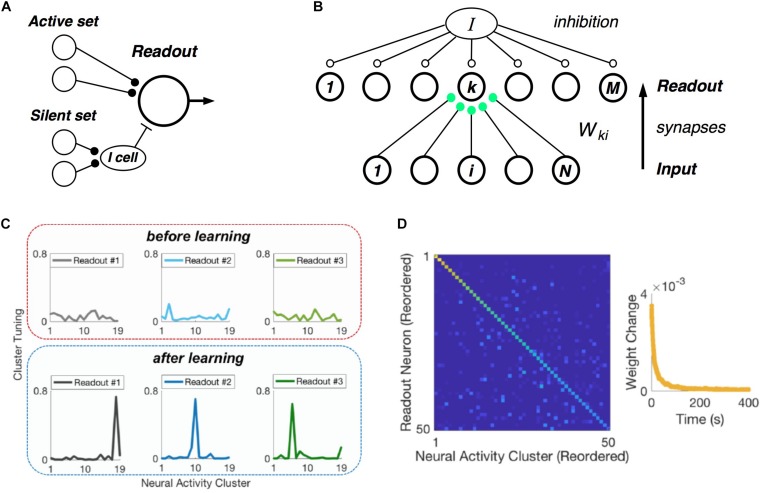
Learning clusters in the input population. **(A)** Simple model of a readout neuron that has excitatory synapses from neurons in the active set of a given cluster and disynaptic inhibition from the silent set. **(B)** Winner-take-all neural (WTA) circuit has a layer of readout neurons with feedforward synapses from the input population having Hebbian plasticity, along with global inhibition. **(C)** Example tuning curves for three readout neurons before (top) and after learning (bottom) on measured retinal population activity. Each panel plots the spiking probability of readout neuron as a function of the neural activity cluster present in the input. **(D)** Confusion matrix for the WTA circuit after learning, displaying readout efficacy for all readout neurons and clusters (color scale). Right*:* Mean absolute synaptic weight change plotted as a function of time during learning.

Central to the hypothesis that neural populations use a cluster code is the requirement that real, downstream neural circuits should be able to readout cluster identity from their input population – namely, that such a neural code is easily “learnable.” In formulating our picture of clusters as comprised of neuronal communities, we realized that there exists an exceptionally simple decoding rule that can identify communities, and hence clusters (Figure 7A) (Loback et al., 2017). Each neuron in the active set should make an excitatory synapse onto the readout unit. Then if at least *K*^*min*^ such neurons fire, the readout cell could spike. In addition, each member of the silent set of neurons would drive a local inhibitory cell, which would feed forward onto the readout neuron. This inhibitory cell would veto the readout unit if any of its inputs were active, thus enforcing the requirement that all members of silent set are silent.

Of course, this decoding mechanism only works with the proper choice of synaptic weights. Thus, the crucial issue is whether there exists a biologically plausible neural circuit with synaptic plasticity rules that can learn the proper weights. To this end, we formulated a layer of readout neurons, which receive spikes from the input population via feedforward synapses, and which have global inhibition (Figure 7B). The feedforward synapses have Hebbian plasticity, and there is homeostatic plasticity in each readout neuron (Loback and Berry, 2018).

When we presented this circuit with measured retinal spike trains, the readout neurons developed cluster tuning, meaning that they responded strongly to any population input pattern within one cluster and weakly to all other activity patterns (Figure 7C). Cluster tuning developed, in large part, because of Hebbian synaptic plasticity. However, global inhibition played a key role by causing different readout neurons to specialize for different clusters. In fact, if the readout circuit had moderate redundancy (i.e., two times more readout neurons than input clusters), then all input clusters could be learned (Figure 7D) (Loback and Berry, 2018). This learning process unfolded in real time – i.e., only ∼1% of the dataset was needed to reach the steady-state of the learning process (Figure 6D, right). Finally, homeostatic plasticity of excitability helped readout neurons to encode the probability of occurrence of each cluster.

These basic ingredients are found quite generically throughout the brain. For instance, these computational elements are present within every layer of the neocortex, as well as in the hippocampus, striatum, thalamus, etc. This prevalence suggests that the ability to readout and process the information encoded in clusters of population activity is widely present in the brain, making this population coding principle widely applicable.

As stated earlier (Figure 1), an appealing aspect of this cluster-reading mechanism is that this operation can be repeated within the neocortical hierarchy. When clusters are learned in layer 4, those readout neurons necessarily acquire new feature selectivity, as is observed within the visual cortex, particularly in the ventral stream (Ungerleider and Haxby, 1994; Hubel, 1995). Because clusters are defined by correlation among neurons, neurons that readout different clusters will necessarily have low correlation. This property is consistent with the pattern of correlation versus layer within the primary visual cortex: namely, pairwise correlations are low in layer 4, but are considerably larger in all other layers (Hansen et al., 2012). Then, if neural activity from layer 4 is nonlinearly recombined in layer 2/3, as has been proposed for the emergence of complex cell receptive fields (Hubel and Wiesel, 1962), a new and stronger pattern of correlation would be created among neurons. As long as the total correlation in layer 2/3 was sufficiently strong to create a cluster code, then this new pattern of correlation layer 2/3 would result in a new set of clusters. When these signals ascend to the next stage in the cortical hierarchy, layer 4 can again learn these clusters, and the whole operation would be iterated. Thus, these alternating computations can create a system that learns increasingly complex visual features with no supervision (Figure 1).

## Discussion

We have shown that under a wide variety of conditions, the population activity patterns of retinal ganglion cells are structured into a discrete set of clusters. This clustering results from heterogeneous correlations among neurons that are sufficiently strong. Because pairwise correlations mostly induce redundancy in the representation of visual information, clustering can also be interpreted as a consequence of sufficient redundancy of the population code. This redundancy in turn allows clusters to have error correcting properties, due to the fundamental relationship between redundancy and error correction (McKay, 2003; Mezard and Montanari, 2009). Finally, this level of redundancy also provides an answer to the question of why the retina has so many ganglion cell types – namely, this heterogenous over-representation of the visual world is required in order to create a cluster code.

Redundancy, of course, reduces the total information encoded by a population of neurons, at least when compared against a hypothetical independent population that is matched to have the same firing rates for each neuron (Schneidman et al., 2003a). Redundancy in neural codes seems to emerge from the sheer number of neurons employed by local circuits to represent information. For instance, the ganglion cell population in the tiger salamander retina has a spatial coverage factor of ∼60 (Segev et al., 2006) and an estimated population redundancy of ∼10-fold (Puchalla et al., 2005). On the other hand, if one analyzes only a single ganglion cell type, such as the parasol cell in the monkey, then the redundancy of this subpopulation is only ∼1.2 (Shlens et al., 2009). While the population redundancy of cortical circuits has not been estimated, they use a much larger number of neurons than subcortical circuits. For instance, the retina of humans has ∼1 million ganglion cells while the primary visual cortex has ∼1 billion neurons (Barlow, 2001). Thus, the population redundancy of human V1 is expected to be orders of magnitude higher than the retina.

This strategy of using so many neurons to represent information has several consequences. First, it suggests that the total population can routinely and without any fine-tuning represent *all* of the incoming sensory information. In this sense, the intuition that a redundant population has “less” information than an efficient, independent population is somewhat off the mark, because a highly redundant local circuit actually represents all of the incoming information, anyhow. A second related point is that the goal of achieving a population code with statistically independent information is probably not realistic. To see this, let’s imagine a thought experiment in which a local neural circuit starts with fewer neurons than its input fibers and then begins to add more neurons. As more and more neurons are added, it will become increasingly difficult to create new neurons whose activity is statistically independent of all the other neurons. And as the number of neurons grows even larger and reaches the number found in real local circuits, this goal may become mathematically impossible. In this sense, the intuition that population codes should strive for independent activity is also off the mark. Third, a population code with high enough over-representation is clearly not efficient, at least in terms of representing Shannon information using few neurons or low energy cost. Thus, the “guarantee” of representing all incoming information without needing any circuit-level adjustments is perhaps more valuable than an efficient code that fails in practice to represent some incoming information. Fourth, high over-representation also suggests that these codes must have evolutionary value, as mutations that create local circuits with fewer neurons should be relatively easy to arise.

At the same time, coding information using only the cluster index will reduce the entropy of the population code substantially and hence will also tend to reduce the mutual information. In fact, we estimated that the cluster index alone represented only ∼25% of the information encoded by populations of retinal ganglion cells (Prentice et al., 2016). Another way of viewing this property is to observe that there are very many population activity patterns that fall into the same cluster, giving rise to high entropy within a single cluster (roughly 10 bits for clusters with high spike count (Prentice et al., 2016)). Given this high entropy, it is likely that additional information can be represented by the pattern of activity within a given cluster. More specifically, the set of all activity patterns within the same cluster vary considerably in their overall spike count, *K*. This spike count variable thus has the potential to represent a different kind of information than the cluster index. For instance, the cluster index could represent one of many possible sensory features, while the spike count could represent the *contrast* of that feature. Alternatively, higher spike count could represent higher *certainty* of that sensory feature being present. While these possibilities have not yet been investigated, such hypotheses do point to a way in which the principle of cluster coding could be unified with ideas about probabilistic population codes.

Our notion of a cluster code is similar to the idea of a “thesaurus” of population codewords that are grouped together according to how similar are the stimuli that they encode (Ganmor et al., 2015). We view these two ideas as complementary. In our case, we group activity patterns together according to their statistics and find that clusters encode unique stimuli, while in this other approach, activity patterns that encode similar stimuli are found to be local and compact in response space. The fact that similar structure emerges using two different approaches strengthens the evidence for its validity. We note that one advantage of the present formalism is that these clusters can be learned and read out by downstream neural circuits as the animal explores its environment. In contrast, the thesaurus formalism requires many exact repeats of the stimulus in order to calculate its distance metric, making it is less clear how an animal can implement this clustering approach in real time.

Additional evidence for the organization of neural activity patterns into a discrete set of clusters comes from an alternative analysis that introduced a perturbation to the energy landscape around one reference activity pattern (Huang and Toyoizumi, 2016). The authors found a sharp transition in the Hamming distance between activity patterns as a function of the strength of the perturbation. This implies that there are regions of neural activity space with a high density of patterns separated by regions with a low density. Furthermore, the silent state played a special role as a “hub” that was connected by high state density to most activity patterns, consistent with our picture of the silent state at the peak of a probability mountain with ridgelines radiating down in different directions. This analysis was carried out on a retinal dataset with many repeats of a short movie clip; it will be interesting to see how it generalizes to the case of a broad stimulus ensemble without repeats.

### Cluster Coding and Sparseness

In our experiments with retinal ganglion cell populations, we observed that population activity was highly sparse (Berry et al., 2019). One consequence of this sparseness was the overall geometry of the probability landscape – specifically, the fact that the most common activity pattern was the all-silent state. If the population code were not sparse, then this would not necessarily be true. Furthermore, the ridge-like shape of clusters might also require a sufficiently sparse code. In particular, if we consider the limit where the stimulus ensemble consists of a small number of highly repeated stimuli that drive neurons, then population code will tend to be dense (Schwartz et al., 2012). In this case, the probability landscape breaks into a discrete set of local peaks corresponding to each stimulus (Loback et al., 2017), and these clusters look more like Gaussian distributions than ridges. Furthermore, the most common activity pattern would be related to which stimuli were most common. However even in the limit of stimulus ensembles with a dense population code, the clustered limit will still be present, if the correlations among neurons are strong enough.

As mentioned before, the high redundancy of most population codes makes them not efficient, at least in terms of encoding maximal mutual information per unit of coding cost (such as entropy, energy, or number of neurons). However, it is still possible that population codes are efficient given a more extensive set of requirements or constraints. Here, we have proposed that one of those requirements might be for the code to be learnable, that is, to have well-defined clusters. In our thermodynamic language, this means that the population code must have a peak in the heat capacity that is equal to or greater than unity (the “temperature” of the real neural population), *T*^∗^ ≥ 1. Because this requirement is an inequality, there will be a large set of such possible population codes. But here we have suggested an optimization principle for cluster codes – namely, that such codes achieve maximal information encoded by the cluster index, while maintaining well-defined clusters. Intuitively, we expect that clusters codes with maximal cluster information will tend to have a critical temperature, *T*^∗^ ∼ 1. Thus, this efficiency principle is quite similar, in practice, to the proposal that population codes exist precisely at the critical point. However, this notion of efficient cluster codes does not require the same precise fine-tuning that the notion of criticality typically entails.

### Cluster Coding and Criticality

What is the relationship between an efficient cluster code and the critical state? The theory of critical phenomena was developed in statistical physics to relate well-known microscopic interactions (such as the electrostatic force or Coulomb’s Law) to macroscopic properties of materials. Because macroscopic samples of materials have a very large number of atoms (roughly Avogadro’s number, *N*_*A*_ ∼ 6⋅10^23^), it has been natural to make the approximation that *N* → ∞, known as the “thermodynamic limit.” Strictly speaking, the critical state only exists in this limit. Consequently, the critical state has extreme and precise mathematical properties, such as a divergence in the susceptibility of the system to perturbation (such as the heat capacity) and long-range correlations that decay as a power-law rather than exponential function of distance.

There are two main challenges in relating ideas about statistical criticality to population neural codes. First, the retina is not a physical system at equilibrium, but is instead an energy-dissipating system driven by an external stimulus. With due care in interpretation, it is possible to use the maximum entropy framework and formalisms of statistical mechanics when the retina is stationary (Tkačik et al., 2015). The non-equilibrium nature of the system can, however, also introduce behaviors that are surprising, for example Zipf’s Law – a power-law distribution over microscopic states (Schwab et al., 2014; Tkačik et al., 2015; Humplik and Tkačik, 2017). Second, we need to understand what properties are expected for a finite-sized neural population. Because the population coding unit has hundreds to thousands of neurons, the thermodynamic limit might not be a good approximation. Finite-size effects convert the susceptibility’s divergence into a peak; that is why we direct attention to the peak in the heat capacity of the neural population (Figure 2). Finite size will also cause deviations from Zipf’s Law and possibly reduce the range of correlations, but both of these effects have been less well studied and predictions from theory are unclear. Nevertheless, physicists have productively explored phase-transition-like behavior in systems far from the thermodynamic limit before, such as in proteins that consist of chains only hundreds of amino-acids long, yet nevertheless show clear transitions between definable states (Schnabel et al., 2011).

To investigate the role of finite-size effects, we varied the size of retinal ganglion cell populations that were analyzed together. We found that the peak in the specific heat (the heat capacity per neuron) increased and moved closer to *T* = 1 as we analyzed larger populations. One could interpret this as evidence that the entire ganglion cell population might be in the critical state and therefore endeavor to extrapolate the trends in the specific heat to larger population size. However, one difficulty with this extrapolation is that larger populations would have ganglion cells spaced farther apart, which would have systematically lower pairwise correlations (Nonnenmacher et al., 2017). Thus, a naïve extrapolation is not valid, and instead experiments that measure much larger populations are needed.

Most importantly, it is unclear what would be the relevance of this kind of extrapolation for understanding the neural code. This is because the entire population of ganglion cells never converges onto a single neuron or group of neurons downstream. Instead, there is a spatial map, where local groups of ganglion cells synapse densely onto local regions of downstream circuits, in a convolutional fashion. Thus, it seems more relevant to ask what are the properties of these local groups of ganglion cells, which is what we define as the *population coding unit*. The population coding unit has a size of ∼200 ganglion cells, which we can already measure and analyze. This analysis reveals that the peak in the heat capacity is slightly above the state of the real system, *T*^∗^ > 1, such that the population is in a *subcritical* state. This view is buttressed by the fact that we already know that pairwise correlations between ganglion cells are ***not*** long-range – namely, they decay substantially outside of the population coding unit (Segev et al., 2006; Pitkow and Meister, 2012). Thus, our interpretation is that the population code of retinal ganglion cells is in a subcritical state, that we refer to as the glassy state.

There are several open questions concerning the relationship between criticality and the cluster code, among which we briefly highlight two. First, we do not know how to fully identify the regimes of measurable statistics of neuronal activity (such as mean firing rates, distribution of pairwise correlations, neuronal population size, etc.) where the high-capacity close-to-critical cluster code would appear. Second, we have presented a qualitative argument whereby frustration generates a ridge-like probability landscape and closeness to criticality assures high-capacity use of the resulting clusters; what we lack, however, is a cleaner mathematical understanding of how these two ingredients combine to the capacity of the resulting neural code.

### Regularization of Population Activity

An important parameter of all our analyses is the choice of a timebin for discretizing spike trains. There are three fundamental criteria that influence this choice. First, there is the temporal precision of spike trains. If one selects a timebin much smaller than the temporal precision, then the additional detail revealed is mostly noise, making this choice impractical. Retinal and LGN spike trains have a temporal precision of ∼10–20 ms under most visual conditions (Berry et al., 1997; Kara et al., 2000; Uzzell and Chichilnisky, 2004; Butts et al., 2007). A second factor is the time scale of noise correlation, as one prefers for the timebin to capture most of this correlation between neurons, so that the combinatorial nature of the code is properly accounted for. For the retina and LGN, the most common form of noise correlation is an excess of spikes between two neurons within a timescale of ∼10 ms (Mastronarde, 1989; Brivanlou et al., 1998; DeVries, 1999). The third, and perhaps most important, factor is the timescale with which downstream neurons will process incoming spike trains. On a biophysical level, the cell membrane imposes low pass temporal filtering, which is typically in the range of ∼10–20 ms. Furthermore, synaptic input currents have their own timescale, typically dominated by the dynamics of ligands binding to receptors or to the dynamics of second messenger cascades. The fastest synaptic currents are found for neurotransmitters, like glutamate and GABA, which have timescales of ∼5–10 ms. Taken together, we can see that all of these factors point to a single “correct” choice for the timebin, namely ∼10–20 ms. Varying the time bin within this range does not cause qualitative differences in the results of our analysis, and so we have settled on a 20 ms timebin (This choice provides slightly more information per bin but less information per unit time). Given this choice of timebin, our analysis of the population code should be considered as a version of a “temporal code” (Rieke et al., 1996).

We have also chosen to binarize spike trains within a single timebin, meaning that we treat one or more spikes as the same coding symbol. This choice is strongly driven by technical tractability, because MaxEnt models become far more unwieldy if a single neuron can have 3+ coding symbols in a single timebin. But because we discretize with a small timebin, it is relatively rare to witness more than one spike in a bin. More specifically, we have found that ignoring multiple spikes in a bin has a fairly small effect on both the information encoded by neurons and the ability to discriminate among stimuli (Schwartz et al., 2012).

Another important issue is the fact that there are correlations in spike trains across multiple timebins. One of the best ways to capture this form of correlation is to discretize into spike “words,” which are formed by concatenating multiple timebins (Strong et al., 1998; Puchalla et al., 2005). For the retina and LGN, temporal correlations typically extend out to ∼100 ms, which is also the timescale of the temporal kernel of individual neurons. This implies forming spike words with ∼5 binary digits might reveal some additional structure in the population code. In a previous study using HMMs, we found that temporal transition matrix was dominated by self-transitions, meaning that the same cluster tended to persist for ∼3–5 consecutive timebins (Prentice et al., 2016). This result implies that temporal correlation had a fairly minor overall effect on the population code. On the other hand, another study that analyzed the thermodynamics of retinal populations found that simply including correlation in the overall spike count, *K*, out to 4 timebins substantially increased the peak in the heat capacity and shifted it closer to *T* = 1 (Mora et al., 2015). This result implies that the retinal population might be significantly closer to the critical state, if temporal correlations were taken into account.

### Retina Versus Cortex

Several of the arguments that imply that the retinal population code is in a sub-critical state do not necessarily apply to the cortex. First, the population coding unit is larger in the cortex. If we similarly define the population coding unit as the number of neurons that project onto any single readout neuron in the cortex (Buzsaki, 2010), then we get a unit size of ∼1000 neurons. Populations of this size may be substantially closer to the thermodynamic limit than retinal ganglion cell populations. Second, pairwise correlation between cortical neurons extends over much larger distances (Smith and Kohn, 2008) and even exists between neurons in different cortical areas (Pesaran, 2010; Raichle, 2015). This pattern of correlation is much longer range than in the retina, even if the specific function of distance is not precisely a power law. This extensive pattern of cortical correlation also relates to the notion of dynamic criticality: namely, the idea that the critical state may help to propagate information across the entire size of the system without requiring axons to reach this far (Traub et al., 1996; Shew and Plenz, 2013). For these reasons, the assumption that cortical populations exist in the critical state might effectively be a very good approximation. This is an important empirical question for future studies to address, as are analogous questions for other brain regions.

At the same time, it appears likely that cortical populations have well-defined clusters. First, larger population coding units in the cortex contribute to clustering, as we have found that analyzing larger populations leads to a sharper peak in the heat capacity (Figure 2) as well as more clusters identified by the HMM (Prentice et al., 2016). Second, despite some controversy (Ecker et al., 2010), pairwise correlations in the cortex appear to be of roughly similar strength as those found in the retina (*r*_*sc*_ ∼0.05 in 20 ms timebins; Smith and Kohn, 2008; Hansen et al., 2012), at least in the superficial layers. In fact, we found in an initial study that the HMM could identify well-defined clusters in populations of neurons recorded from layer 2/3 of the primary visual cortex (Li et al., 2019).

### Cluster Coding for Motor Systems

Most of the focus of this article has been on sensory systems. However, our analysis suggests that neural populations in all areas of the cortex may have sufficient correlation to be structured into a cluster code. How does this idea apply to motor areas of cortex?

The actual range of movement employed by animals is typically low-dimensional in the space of all possible joint and limb degrees of freedom, which represents a form of redundancy of movement patterns compared to total movement capacity. This restricted dimensionality implies that neural populations in motor systems will also be redundant (Gao and Ganguli, 2015), as has been directly observed in the motor cortex (Narayanan et al., 2005; So et al., 2012). Having a cluster code in motor systems has potential benefits. First, clusters can emphasize particular movements that have been useful in the past, so that a wide range of motor planning activity can activate that movement. Second, clusters can enforce a form of motor “grammar,” in which certain combinations of movement are not allowed, by keeping such dysfunctional combinations in separate clusters. Of course, movements can be made with a continuum of speeds and exact trajectories. But recall that the high entropy of neural activity patterns within a cluster has the potential to jointly encode continuous variables. Thus, there is no essential mismatch between a discrete set of clusters of neural activity and the continuous range of possible movement.

In any case, direct demonstration that neural activity is structured into a cluster code is lacking in most brain areas, and as such this empirical question represents a major future direction of study.

## Data Availability Statement

Publicly available datasets were analyzed in this study. This data can be found here: https://datadryad.org/resource/doi:10.5061/dryad.1f1rc, https://dataspace.princeton.edu/jspui/handle/88435/dsp016d5700272.

## Ethics Statement

The animal study was reviewed and approved by the IACUC Committee, Princeton University.

## Author Contributions

MB supervised the original research and drafted the text. GT carried out the original research, supervised the research, and edited the text.

## Conflict of Interest

The authors declare that the research was conducted in the absence of any commercial or financial relationships that could be construed as a potential conflict of interest.

## Footnotes

1 Despite their similarity to the Boltzmann distribution, maximum entropy models *do not* automatically imply that the system being described is a physical system in thermodynamic equilibrium; applied and interpreted carefully, maximum entropy models can describe driven, out-of-equilibrium systems in stationary state (like in the case of neural networks) (Tkačik et al., 2015).

2 Interestingly, in the low-temperature limit, statistical physics of spin-glasses uses a mathematically identical decomposition of the probability distribution into a weighted sum of modes (referred to as “pure states”), as in Eq. 4 (Mezard et al., 1987).
